# Faba Bean (*Vicia faba* L. *minor*) Bitterness: An Untargeted Metabolomic Approach to Highlight the Impact of the Non-Volatile Fraction

**DOI:** 10.3390/metabo13080964

**Published:** 2023-08-21

**Authors:** Adeline Karolkowski, Emmanuelle Meudec, Antoine Bruguière, Anne-Claire Mitaine-Offer, Emilie Bouzidi, Loïc Levavasseur, Nicolas Sommerer, Loïc Briand, Christian Salles

**Affiliations:** 1 Centre des Sciences du Goût et de L’Alimentation, CNRS, INRAE, Institut Agro, Université de Bourgogne, F-21000 Dijon, France; adeline.karolkowski@inrae.fr (A.K.); antoine.bruguiere@u-bourgogne.fr (A.B.); anne-claire.offer@u-bourgogne.fr (A.-C.M.-O.); 2 Groupe Soufflet-Invivo, F-10400 Nogent-sur-Seine, France; llevavasseur@invivo-group.com; 3 SPO, Université de Montpellier, INRAE, Institut Agro, F-34000 Montpellier, France; emmanuelle.meudec@inrae.fr (E.M.); nicolas.sommerer@inrae.fr (N.S.); 4 INRAE, PROBE Research Infrastructure, PFP Polyphenol Analysis Facility, F-34060 Montpellier, France; 5 Vivien Paille (Groupe Avril), F-59300 Valenciennes, France; ebouzidi@vivienpaille.fr

**Keywords:** pulses, faba beans, off-flavours, bitterness, sensory analysis, UHPLC-HRMS, metabolomic approach

## Abstract

In the context of climate change, faba beans are an interesting alternative to animal proteins but are characterised by off-notes and bitterness that decrease consumer acceptability. However, research on pulse bitterness is often limited to soybeans and peas. This study aimed to highlight potential bitter non-volatile compounds in faba beans. First, the bitterness of flours and air-classified fractions (starch and protein) of three faba bean cultivars was evaluated by a trained panel. The fractions from the high-alkaloid cultivars and the protein fractions exhibited higher bitter intensity. Second, an untargeted metabolomic approach using ultra-high-performance liquid chromatography–diode array detector–tandem–high resolution mass spectrometry (UHPLC–DAD–HRMS) was correlated with the bitter perception of the fractions. Third, 42 tentatively identified non-volatile compounds were associated with faba bean bitterness by correlated sensory and metabolomic data. These compounds mainly belonged to different chemical classes such as alkaloids, amino acids, phenolic compounds, organic acids, and terpenoids. This research provided a better understanding of the molecules responsible for bitterness in faba beans and the impact of cultivar and air-classification on the bitter content. The bitter character of these highlighted compounds needs to be confirmed by sensory and/or cellular analyses to identify removal or masking strategies.

## 1. Introduction

In the context of climate change, it seems appropriate to reconcile the economic, social, and environmental impacts of human activities. The transition to a more plant-based diet, in particular in pulses, thus appears to be one of the main levers for improving human health and the sustainability of food systems [1]. For example, faba bean (*Vicia faba* L. *minor*) ingredients are increasingly used in the formulation of food products due to agronomic, environmental, nutritional, and functional interests [2,3]. Despite the great interest in pulses, consumer acceptability remains low due to off-flavours, such as off-notes, bitterness, and astringency [4]. However, faba beans are still of sensory interest. They are not astringent and have a lower intensity of off-notes than other pulses, such as peas or soybeans [5,6]. Research on the off-flavours of faba beans has focused on the involvement of volatile compounds in off-notes [7,8]. In addition, a few studies have been carried out on the molecules responsible for the bitterness of pulses, but they were mainly conducted on peas and soybeans [9].

Many compounds from plant defence metabolisms could be responsible for pulse bitterness [9]. Recent studies have highlighted the role of saponins, phenolic compounds, peptides, lipids, and lipid oxidation products in the bitterness of peas [10,11,12,13]. Concerning lupins, bitter perception should be more related to specific alkaloids and tannins [14]. Such research is similarly scarce for the bitter compounds in faba beans. In general, only the profile of non-volatile compounds has been studied in faba beans without making a link to their sensory perception. The main identified metabolites belonged to alkaloids, phenolic compounds, saponins, jasmonates, organic acids, and amino acids [15,16,17,18,19,20,21,22]. It has been suggested that the presence of free phenolic compounds, vicine and convicine was linked to bitterness in faba bean ingredients (flour, protein concentrate, and isolate). Moreover, saponins and tannins have been proposed to play a minor role in bitter perception [8]. This observation was partly confirmed by a recent study that demonstrated that the concentration of saponins in faba beans was too low to contribute to their bitterness. However, vicine (one of the major alkaloids) activated one of the 25 human bitter taste receptors (TAS2Rs), and its concentration in faba beans should partly account for bitterness [23].

The aim of this study was to determine the non-volatile compounds that could contribute to faba bean bitterness by linking chemical and sensory data. To this end, three cultivars and three fractions (flour (F), starch fraction (S), and protein fraction (P) (concentrate)) of faba beans were studied. First, the sensory bitterness of the fractions was evaluated by a trained panel. Second, the non-volatile profiles of the different samples were characterised by a metabolomic approach using ultra-high-performance liquid chromatography–diode array detector–tandem–high resolution mass spectrometry (UHPLC–DAS–HRMS). Third, the metabolomic and sensory data were correlated, and the compounds positively correlated with bitterness were tentatively identified. All the selected sensory and physico-chemical methods have already been validated and used to make a link between the chemical composition of mushrooms or peas with their sensory attributes [10,24].

## 2. Materials and Methods

### 2.1. Faba Bean Fractions

Three cultivars (C1, C2, and C3) of faba beans (*Vicia faba* L. *minor*) were studied. Mature seeds were harvested in 2020 and provided by Soufflet Agriculture (Groupe Soufflet-Invivo, Nogent-sur-Seine, France). C1 was cultivated under organic conditions. C1 and C2 were richer in alkaloids (vicine and convicine) than C3. The seeds were dehulled, ground, and air-classified by an external laboratory (SAS IMPROVE, Amiens, France). The F, P, and S fractions obtained for each cultivar were vacuum-packed in a glass container and stored at −20 °C before analysis.

### 2.2. Sensory Analysis

Faba bean fractions were incorporated into a gel made of xanthan gum and water. Twenty-one trained panellists (35–73 years old, 12 females and 9 males) evaluated the bitterness of the 9 gels along a linear scale (from 0 (absence) to 10 (very intense)). A solution of caffeine (0.5 g/L) was used as an external reference for bitterness. Additional details on the gel formulation and sensory profiling can be found elsewhere [23,25].

### 2.3. Non-Volatile Compound Extraction

Extraction of the non-volatile compounds was adapted from previous protocols [10,26]. For each fraction, 100 mg of sample was extracted twice with 5 mL of a methanol (Biosolve Chimie, Dieuze, France) and formic acid (Biosolve Chimie, Dieuze, France) mixture (99/1 *v*/*v*) under a 1 h constant agitation at room temperature. The suspensions were kept at −20 °C for 20 min and centrifuged (4500 rpm, 20 min, 4 °C). Then, the supernatant was evaporated at 35 °C using a centrifugal evaporator (EZ-2 Plus Evaporating System, Genevac, Ipswich, UK). After evaporation, the samples were solubilised in 200 µL of a methanol, Milli-Q^®^ ultrapure water (Merck Millipore, USA), and formic acid mixture (49.5/49.5/1 *v*/*v*) before being centrifuged (15,000 rpm, 15 min, 4 °C). The supernatants were collected through a 0.22 µM PTFE (polytetrafluoroethylene) filter, placed in HPLC vials and stored at −80 °C in darkness until analysis. The samples were prepared in triplicate.

### 2.4. Non Volatile Compound Untargeted Analysis

The extracts were analysed by UHPLC (Vanquish, Thermo Scientific, Waltham, MA, USA) using an Acquity UPLC HSST3 C18 column (100 mm × 1 mm i.d., 1.7 µm; Waters, Saint-Quentin-en-Yvelines, France). The mobile phase consisted of (A) water–formic acid (99/1 *v*/*v*) and (B) acetonitrile–water–formic acid (79.5/19.5/1 *v*/*v*/*v*). The gradient program with a flow rate of 0.22 mL/min was set as follows: 1–1.5 min A/B, 98/2%; 4.5–7 min A/B, 88/12%; 12 min A/B, 76/24%; 15 min A/B, 72/28%; and 16 min A/B, 40/60%. The column and injector temperatures were maintained at 35 and 10 °C, respectively. The injection volume was 0.5 µL. The UHPLC system was coupled with a DAD (UV–visible diode array detector) covering the full range of acquisition (190–600 nm) and an HRMS (Orbitrap Exploris^TM^ 480, Thermo Scientific, Waltham, MA, USA) equipped with a heated electrospray ionisation probe. HRMS was operated in both negative and positive ion modes. The parameters for the ion source were as follows: ion transfer tube temperature—280 °C; voltage (+)—3500 V; voltage (−)—2500 V; sheath gas—40 a.u.; auxiliary gas—10 a.u.; sweep gas—2 a.u.; vaporiser temperature—300 °C; mass range—100–1800 Th, resolution—at *m*/*z* 240,000 and 480,000. The sample sequence was adapted from a previous method [27]. It included extracts from S, F, and P fractions, a quality control (QC, which was a mix of all samples) sample, and blank. Samples were injected in biological triplicate and in a random order. After equilibration, the sequence started with 3 injections of a blank, followed by 5 injections of QC samples. The blank and QC sample were then injected every 7 real samples. The sequence ended with 1 injection of QC sample and 1 injection of blank. Xcalibur^TM^ 4.4 (Thermo Scientific, Waltham, MA, USA) was used for instrument control, data acquisition, and data analysis.

### 2.5. Metabolomic Analysis

Raw data from HRMS were processed with Compound Discoverer™ (v 3.2.2.421, Thermo Scientific, Waltham, MA, USA), which facilitated peak recognition and dereplication of raw data (retention time alignment, adducts, and isotopic peak clustering). Details about the workflow are available in the Supplementary Materials. Then, these data were correlated (Pearson correlation) with the bitter intensity of the faba bean fractions to identify those that were positively correlated. The tentative identification of the positively correlated compounds was based on ultra-high-resolution mass spectrum, mass accuracy below 1 ppm, characteristic MS/MS fragmentation, UV spectrum, and retention time available in the literature. In particular, the isotope-ratio at ultra-high resolution of ^15^N vs. ^13^C and ^18^O vs. 2x^13^C and ^34^S was used to determine the chemical formula of the studied compounds (FreeStyle 1.5, Thermo Scientific, Waltham, MA, USA).

### 2.6. Statistical Analysis

Analyses were performed using XLSTAT (Addinsoft, Paris, France). For the bitter intensity, a one-way analysis of variance (ANOVA) was performed, and significant differences were evaluated by Tukey’s honest significant difference (HSD) post hoc test (*p* < 0.05). Linear and logarithmic Pearson correlations (α = 2.5%) were used to determine the relationships between the non-volatile compound areas, in both negative and positive modes, and the bitter intensity of the 9 fractions. Only compounds positively correlated with bitterness were then tentatively identified. In addition, a principal component analysis (PCA, Pearson correlation) was also carried out to visually explore the differences in the non-volatile compound profile of the studied samples.

## 3. Results and Discussion

### 3.1. Bitterness of the Faba Bean Fractions

**Figure 1:**
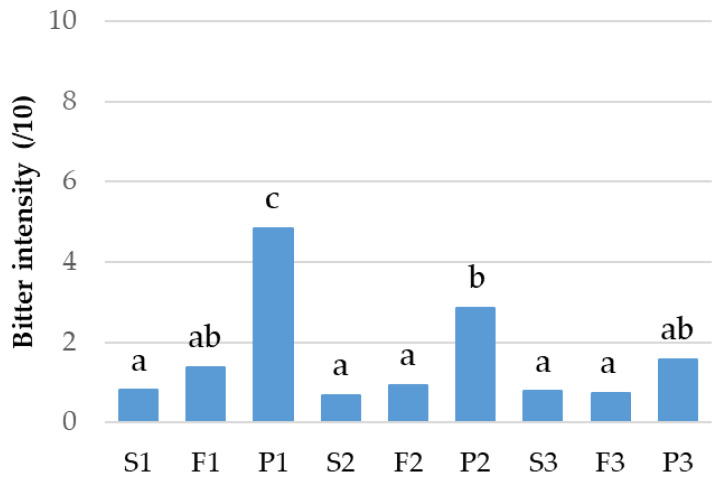
Bitter intensity (over 10) of the 9 fractions evaluated by a trained panel. Significant differences are indicated by different letters (Tukey’s HSD test, α = 5.0%). S: starch fraction; F: flour; P: protein fraction—the number after the fraction corresponds to the cultivar (1, 2, or 3).

The bitter taste of the nine gels was evaluated by a trained panel (Figure 1). No significant difference in the bitter intensity was observed for the three flours. However, the P fractions, except for P3, were perceived as more bitter than the F and S fractions. P1 was also perceived as more bitter than P2 and P3. These sensory data were correlated with those from the untargeted metabolomic approach to highlight potential bitter compounds in faba bean fractions.

### 3.2. Tentative Identification of Non-Volatile Compounds Correlated with Faba Bean Bitterness

**Figure 2:**
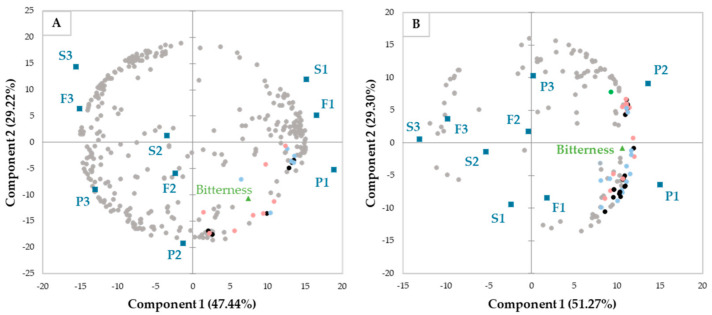
Biplot representation of the PCA (centred reduced variables, Pearson correlation, α = 5.0%) of the detected compound areas in the negative (**A**) or positive (**B**) modes and the perceived bitterness (as a supplementary variable in light green) of the 9 faba bean fractions. The compounds positively correlated with bitterness are related to variables in blue for the linear model, in dark green for the logarithmic model and in pink for both the linear and logarithmic models, whereas the black highlighted compounds correspond to the mis-dereplicated data of the positively correlated compounds. S: starch fraction; F: flour; P: protein fraction—the number after the fraction corresponds to the cultivar (1, 2, or 3).

A list of 355 and 155 potential compounds was established in negative and positive modes, respectively. In the negative mode, 14 non-volatile compounds were positively correlated with bitterness using linear and/or logarithmic correlations, whereas in the positive mode, 31 compounds were positively correlated with bitterness. Only three compounds were detected and positively correlated with bitterness in both positive and negative modes. Figure 2 shows the different non-volatile compounds resulting from the metabolomic analysis of the fractions and those that were positively correlated with bitterness (variables in blue for linear model, variables in dark green for logarithmic model and variables in pink for both models). Few adducts or fragments of these compounds were also positively correlated with bitterness. Those adducts should have been dereplicated by the workflow used but were not. An expert overlooked of the features after dereplication was carried out, and they were easily identified. As there were few mis-dereplications, the workflow was not modified. Those mis-dereplicated compounds were not further considered for this analysis; however, they were represented by black variables on the PCA.

Then, the positively correlated compounds were tentatively identified by comparing the UV spectrum and the MS/MS spectra from the literature. Moreover, the occurrence of tentatively identified compounds in faba beans and/or other pulses/plants was also specified. For all the proposed identifications, the use of high-mass accuracy (<1 ppm) and ultra-high-resolution MS spectra (R = 480 k at *m*/*z* 200) of the isotopic profile allowed unique and unambiguous molecular formulas. The correlated compounds are presented in Table 1. They are distributed into the following chemical classes: alkaloids, amino acids, phenolic compounds, organic acids, other compounds, and unidentified compounds.

#### 3.2.1. Alkaloids

A total of six compounds belonging to the alkaloid family were tentatively identified. Vicine (1) had one absorption band at 274 nm and displayed a major molecular ion at *m*/*z* 305.1093 in the positive mode. The fragment at *m*/*z* 143.0564 in the MS^2^ spectra should indicate the presence of divicine ([C_4_H_6_N_4_O_2_ + H]^+^) and the loss of a hexoside residue (−162). Vicine and convicine are the main alkaloids widely spread in *Vicia faba* [16,28]. Although convicine had been identified in both positive and negative modes, it was not positively correlated with bitterness (linear and logarithmic models). The compounds (**2**)–(**4**) and (**6**) had one absorption band between 270 and 274 nm and exhibited two characteristic fragments of vicine in their MS^2^ spectra: *m*/*z* 305.1093 ([vicine + H]^+^) and 143.0564 *m*/*z*; they were tentatively identified as vicine derivatives. Only the derivative of vicine ((4), *m*/*z* 387.1521 (negative) and 389.1667 (positive)) has already been identified in faba beans [19,21]. It also demonstrated the loss of the C_5_H_8_O moiety in both positive and negative modes that could correspond to a valeric or isovaleric residue according to Kowalczyk et al. (2021) [21]. Moreover, a compound derived from convicine (**5**) was detected at 7.49 min and exhibited a mass of *m*/*z* 388.1361 and 390.1507 in negative and positive modes, respectively. It was characterised by two fragments: *m*/*z* 304.0791 (negative mode) corresponding to convicine and *m*/*z* 142.0264 (negative mode) and 144.0405 (positive mode) corresponding to isouramil [23,28]. The mass difference between the alkaloid derivative and the ion concerning the respective alkaloid was similar (−84) for the compounds (**4**) and (**5**), which also suggested the presence of a valeric or isovaleric residue for convicine derivative (**5**). Many derivatives of alkaloids have been identified in faba beans, even if they did not all correspond to those tentatively identified in this article [16,19,21].

#### 3.2.2. Amino Acids

Many amino acids and their derivatives were detected in positive mode and positively correlated with faba bean bitterness. The compound (**7**), with a mass of *m*/*z* 175.1192 ([M+H]^+^), was also found under a potassium adduct *m*/*z* 213.0747 ([M+2K-H]^+^) and showed a neutral molecular formula of C_6_H_14_N_4_O_2_. The fragments *m*/*z* 158.0926, 130.0975, 116.0707, 112.0870, and 60.0557 were characteristic of L-arginine [29]. The L-arginine ion and the associated fragments were found in the fragmentation of the three following compounds (**8**, **9**, and **17**), suggesting that they were derived from this amino acid. Only N-formyl-L-arginine (**9**) showing the same molecular formula and its fragmentation has already been identified in another plant, the black cohosh [30]. At 1.00 min, the mass of *m*/*z* 198.0762 associated with the fragments *m*/*z* 181.0496, 152.0707, 139.0390, and 135.0441 was related to L-DOPA (**12**) [22]. An isomer of L-DOPA hexoside (**13**) was observed in its protonated (*m*/*z* 360.1286) and stacked (*m*/*z* 719.2506) forms. The fragmentation patterns revealed the loss of a hexoside residue (*m*/*z* 198.0762; −162) and an ammonia moiety (*m*/*z* 181.0495; −17); these observations were consistent with previous results [16,18]. Few isomers have already been identified in faba beans [16,18], but it was not possible to determine which isomer was positively correlated with bitterness. Moreover, three other L-DOPA derivatives were tentatively identified. They exhibited the same characteristic fragments related to L-DOPA, but only the fragmentation of the compounds (**11**) and (**19**) revealed the neutral loss of a hexoside compared to the compound (**20**). However, it was not possible to determine a molecular formula by the isotope-ratio method due to the presence of other signals at the same retention time for the compound (**19**). L-phenylalanine (**16**) and L-tryptophan (**18**) were also tentatively identified, and their fragmentations were in agreement with a previous study [29]. The compound (**15**) exhibited the same fragment *m*/*z* 146.0601 as L-tryptophan, suggesting that it was derived from this amino acid. Concerning the compounds (**10**) and (**14**), their fragments (*m*/*z* 224.0918, 178.0864, and 165.0547) were similar to those of N-acetyl-L-tyrosine [31]. Moreover, their fragmentation patterns revealed the loss of a hexoside residue (*m*/*z* 224.0918; −162); which suggested that these compounds were two isomers of N-acetyl-L-tyrosine hexoside. N-acetyl-L-tyrosine was produced in common bean plants during fungal pathogen attacks [32]. Finally, it is important to note that L-arginine, L-DOPA, L-tryptophan, L-phenylalanine, and L-tyrosine have already been identified in faba beans [16,18,33], which suggested that the tentatively identified compounds were indeed derived from these amino acids.

#### 3.2.3. Phenolic Compounds

Three phenolic compounds, only detected in the negative mode, were positively correlated with bitterness. p-Hydroxybenzoic acid (**21**) was tentatively identified by comparing the accurate MS^1^ and MS^2^ spectra from the literature (obtained on a standard) [10]. The compound (**22**) had two absorption bands at 257 and 293 nm characteristic of phenolic acids [10,34]. The molecular ion at *m*/*z* 315.0721 and the fragment *m*/*z* 153.0194 should indicate the presence of a protocatechuic acid moiety and the loss of a hexoside residue [34]. Concerning the compound (**23**), the band at 282 nm and the fragments from MS^2^ spectra suggested the presence of methylfukiic acid [16,35]. This compound was identified by NMR in *Piscidia Erythrina* L., belonging to the *Fabaceae* family [35]. p-Hydroxybenzoic acid has already been identified in peas [10], whereas protocatechuic acid hexoside and methylfukiic acid were detected out in faba beans [16,17,19,20,21].

The compound (**24**) had two absorption bands at 270 and 340 nm corresponding to the phenolic core and the conjugated system of a flavonoid, respectively [36]. Moreover, its fragment ion at *m*/*z* 287.0551 indicated the presence of a kaempferol moiety. Many kaempferol derivatives have already been observed in faba beans (leaves, pods, and mature seeds) and peas (leaves and protein isolates) [10,15,19,20,21].

#### 3.2.4. Organic Acids

A total of three organic acids were tentatively characterised. The fragmentation patterns in the positive mode of the compound (**25**) were consistent with those of hydroxy aspergillic acid, and this compound has already been identified in brown alga [37]. The compound (**26**) exhibited the fragments *m*/*z* 190.0499, 172.0394, and 144.0444, which should indicate that it was derived from kynurenic acid, which has been identified in olive fruit [38,39,40]. The MS^1^/MS^2^ spectra of the compound (**27**) were characteristic of pantothenic acid hexoside [41,42]. Pantothenic acid has been found in faba beans [16,18], whereas pantothenic acid hexoside has also been detected in chickpeas and tomatoes [41,42].

#### 3.2.5. Terpenoids

A total of two terpenoids were detected. The compound (**28**) was tentatively identified as 8-β-D-glucopyranosyloxy-2,7-dimethyl-2,4-decadiene-1,10-dioic acid by comparing the fragmentation patterns with the faba bean literature [16]. Another terpenoid (**29**) was detected in both negative and positive modes and exhibited the same fragments as dihydrophaseic acid 4’-O-β-D-glucopyranoside; dihydrophaseic acid and its derivatives were detected in faba beans [16].

#### 3.2.6. Other Non-Volatile Compounds

Two compounds were also tentatively identified, but they did not belong to a specific class. First, the compound (**30**) detected at 0.74 min exhibited fragments at *m*/*z* 104.1071, 60.0808, and 58.0657, indicating that it was derived from choline [43], which has already been detected in faba beans [44]. Choline was also tentatively identified in samples with the same MS^2^ spectrum but was not positively correlated with bitterness. Second, the fragments *m*/*z* 315.1812, 191.0564, 161.0456, 149.0456, 143.0347, and 131.0349 indicated that the compound (**31**) should be a geraniol pentoxide hexoside [45]. However, it was not possible to determine the corresponding isomer. Geraniol has already been detected in the volatile content of faba beans [7].

#### 3.2.7. Unidentified Compounds

A total of 11 compounds were not identified among the 45 metabolites positively correlated with bitterness; however, a few comments could be made. The fragmentation patterns of the compound (**35**) revealed the loss of a hexoside moiety (*m*/*z* 321.1010; −162). It was not possible to tentatively identify the compounds (**40**) and (**42**) because their fragmentation data were not available in the literature. The compound (**40**) could be a diterpene glycoside called 19-hydroxycinnzeylanol 19-glucoside, which has already been identified in the leaf of a dicotyledon plant [46]. Concerning the compound (**42**), the MS^1^ spectrum was consistent with those of 4-chloro-oxoindole-acetic acid, which have been detected in faba bean seeds [21]. However, these hypotheses must be verified.

**Table 1:** Tentative identification of the non-volatile compounds tentatively identified and positively correlated with faba bean bitterness (linear and logarithmic models; Pearson correlation, α = 2.5%). The *p*-value is shown in bold when a positive correlation is observed (*p*-value < 0.025).

No.	RT (min)	Linear Model		Logarithmic Model		UV (nm)	Mode	Experi- mental *m*/*z*	Formula (Neutral)	Expected *m*/*z*	Error (ppm)	Main MS/MS Fragment Ions	Compound	RI	Ref.
		**R**	***p*** **-Value**	**R**	***p*** **-Value**										
**ALKALOIDS**															
1	0.86	0.737	**0.024**	0.549	0.126	274	POS	305.1093	C_10_H_14_N_4_O_7_	305.1092	0.10	143.0564 (100)	Vicine	1,2,3,4	[23,28]
2	1.27	0.796	**0.010**	0.598	0.089	274	POS	391.1097	C_13_H_18_N_4_O_10_	391.1107	1.00	305.1099 (1); 143.0564 (100)	Vicine derivative	1,2	
3	6.61	0.83	**0.006**	0.628	0.070	278	POS	613.1988	C_25_H_32_N_4_O_14_	613.1988	0.00	305.1093 (1); 147.0442 (60); 143.0564 (100)	Vicine derivative	1,2	
4	7.61	0.785	**0.012**	0.609	0.082	274	NEG	775.3110 (387.1512)	C_15_H_24_N_4_O_8_	387.1521	0.90	387.1512 (50); 303.0947 (5); 141.0418 (100)	Vicine derivative (ester with valeric/isovaleric acid)	1,2,4	[19,21]
		0.871	**0.002**	0.701	0.035	278	POS	389.1670		389.1667	−0.30	305.1092 (5); 143.0564 (100)			
5	7.68	0.829	**0.006**	0.645	0.060	274	NEG	777.2793 (388.1352)	C_15_H_23_N_3_O_9_	388.1361	0.90	388.1352 (100); 304.0791 (5); 142.0264 (5)	Convicine derivative (ester with valeric/isovaleric acid)	1,2,4	[19,21]
		0.883	**0.002**	0.648	0.059	274	POS	390.1509		390.1507	−0.20	229.1070 (20); 144.0405 (100); 127.0390 (20); 85.0648 (30); 57.0699 (20)			
6	11.56	0.755	**0.019**	0.579	0.102	274	POS	815.3052	C_32_H_46_N_8_O_17_	815.3054	0.20	305.1093 (5); 143.0565 (100)	Vicine derivative	1,2	
**AMINO ACIDS**															
7	0.71	0.736	**0.024**	0.695	0.038		POS	213.0747 (175.1192)	C_6_H_14_N_4_O_2_	175.1201	0.90	175.1192 (100); 158.0925 (20); 130.0975 (10); 116.0707 (30); 112.0869 (5); 60.0557 (20)	L-arginine	1,4	[29,33]
8	0.74	0.885	**0.002**	0.792	**0.011**		POS	292.1979	C_11_H_25_N_5_O_4_	292.1979	0.00	175.1190 (70); 158.0926 (10); 118.0863 (100); 116.0705 (1); 60.0556 (1)	L-arginine derivative	1	
9	0.85	0.846	**0.004**	0.811	**0.008**		POS	203.1138	C_7_H_15_N_4_O_3_	203.1150	1.20	203.1143 (100); 186.0876 (10); 175.1191 (20); 158.0928 (10); 144.0657 (20); 130.0974 (1); 116.0707 (10); 112.0870 (5); 88.0870 (5); 70.0651 (1)	N-formyl-L- arginine	1,6	[30]
10	0.93	0.787	**0.012**	0.765	**0.016**		POS	178.0863 (386.1447)	C_17_H_23_NO_9_	386.1456	0.90	224.0917 (100); 178.0863 (40); 85.0284 (15)	N-acetyl-L-tyrosine hexoside (unknow isomer)	1,4	[16,18]
11	0.96	0.753	**0.019**	0.706	0.033		POS	568.1873 (730.2401)	C_25_H_43_NO_21_	730.2411	1.00	198.0761 (100); 181.0496 (40); 152.0707 (30)	L-DOPA hexoside derivative	1	
12	1.00	0.888	**0.001**	0.815	**0.007**		POS	198.0762	C_9_H_11_NO_4_	198.9761	−0.10	181.0496 (40); 152.0707 (100); 139.0390 (40); 135.0441 (20)	L-DOPA	1,4	[22]
13	1.02	0.797	**0.010**	0.658	0.054		POS	719.2506 (360.1286)	C_15_H_21_NO_9_	360.1289	0.30	360.1286 (5); 198.0760 (100); 181.0495 (10); 152.0704 (10); 139.0390 (20); 85.0284 (5)	L-DOPA hexoside (unknow isomer)	1,4	[16,18]
14	1.11	0.770	**0.015**	0.736	**0.024**		POS	386.1446	C_17_H_23_NO_9_	386.1456	1.00	224.0918 (100); 178.0864 (40); 165.0547 (1); 85.0284 (10)	N-acetyl-L-tyrosine hexoside (unknow isomer)	1	
15	1.86	0.844	**0.004**	0.728	**0.026**		POS	323.0874	C_14_H_14_N_2_O_7_	323.0874	0.00	146.0601 (100)	L-tryptophan derivative	1	
16	2.19	0.813	**0.008**	0.788	**0.012**		POS	166.0863	C_9_H_12_NO_2_	166.0863	0.00	149.0597 (5); 120.0808 (100); 103.0542 (5)	L-phenylalanine	1,4	[29,33]
17	3.60	0.900	**0.001**	0.717	**0.030**		POS	259.1764	C_11_H_22_N_4_O_3_	259.1765	0.10	259.1766 (100); 242.1499 (20); 200.1279 (10); 175.1191 (10); 158.0925 (20); 112.0871 (1). 116.0705 (1); 70.0651 (5)	L-arginine derivative	1	
18	5.30	0.838	**0.005**	0.794	**0.011**		POS	188.0706 (205.0969)	C_11_H_12_N_2_O_2_	205.0971	0.20	205.0969; 146.0602 (100); 118.0652 (10)	L-tryptophan	1,4	[29,33]
19	9.68	0.734	**0.024**	0.689	0.040		POS	222.0648 (443.1226)	ND	-	-	281.0705 (30); 252.0441 (20); 237.0800 (80); 198.0761 (50); 181.0497 (80); 152.0708 (100); 139.0392 (30); 135.0442 (20); 85.0650 (30)	L-DOPA hexoside derivative	1	
20	11.28	0.734	**0.024**	0.652	0.057		POS	444.1867	C_20_H_29_NO_10_	444.1864	−0.30	229.1071 (20); 198.0761 (100); 181.0496 (70); 152.0706 (60); 139.0390 (10); 135.0440 (10); 85.0648 (30)	L-DOPA derivative	1	
**PHENOLIC COMPOUNDS**															
21	1.57	0.851	**0.004**	0.781	**0.013**		NEG	299.0773	C_13_H_16_O_8_	299.0772	−0.10	137.0244 (100); 93.0345 (30)	p-Hydroxybenzoic hexoside	1,4,5	[10,42,47]
22	2.63	0.808	**0.008**	0.730	**0.025**	257; 293	NEG	315.0721	C_13_H_16_O_9_	315.0722	0.10	153.0194 (50); 152.0114 (100); 109.0295 (30); 108.0217 (40)	Protocatechuic acid Hexoside	1,2,4,5	[16,34,42,47]
23	4.94	0.860	**0.003**	0.842	**0.004**	282	NEG	285.0616	C_12_H_14_O_8_	285.0616	0.00	223.0613 (20); 209.0456 (40); 195.0663 (100); 137.0608 (30)	3′-O-Methylfukiic acid (3-O-methyl (3′,4′-dihydroxybenzyl tartaric acid))	1,2,3,4,5	[16,35]
24	12.80	0.824	**0.006**	0.742	**0.022**	270; 340	POS	595.1658	C_27_H_30_O_15_	595.1657	−0.10	287.0551	Kaempferol derivative	1,2,4,5	[10,15,48]
**ORGANIC ACIDS**															
25	1.07	0.732	0.025	0.735	**0.024**		POS	241.1546	C_12_H_20_N_2_O_3_	241.1547	0.10	241.1546 (100); 242.1585 (10); 196.0965 (1); 168.0365 (1); 128.1069 (5); 84.0444 (5)	Hydroxy aspergillic acid	1,6	
26	2.87	0.907	**0.001**	0.816	**0.007**		POS	305.0768	C_14_H_12_N_2_O_6_	305.0779	1.10	215.0814 (20); 190.0499 (50); 172.0394 (100); 144.0444 (10)	Kynurenic acid derivative	1	[38,39]
27	4.35	0.902	**0.001**	0.843	**0.004**		NEG	380.1552	C_15_H_27_O_10_N	380.1562	1.00	362.1441 (10); 308.1351 (20); 218.1036 (10); 146.0822(90)	Pantothenic acid hexoside	1,5,6	[41,42]
**TERPENOIDS**															
28	6.14	0.778	**0.013**	0.769	**0.016**		NEG	403.1602	C_18_H_28_O_10_	403.1599	−0.30	403.1602 (100); 223.0976 (5); 179.1077 (5); 161.0455 (10); 59.0138 (50)	8-β-D-glucopyranosyloxy-2,7-dimethyl-2,4-decadiene-1,10-dioic acid	1,4	[16]
29	6.25	0.780	**0.013**	0.751	**0.020**		NEG	887.3914 (443.1916)	C_21_H_32_O_10_	443.1923	0.70	281.1314 (1); 237.1497 (5); 219.1391 (10); 161.0454 (10); 101.0244 (70)	Dihydrophaseic acid 4’-O-β-D-glucopyranoside	1,4	[16,19,21]
		0.834	**0.005**	0.781	**0.013**		POS	467.1887 (445.2068)		445.2079	1.10	284.0916 (50); 143.0563 (100)			
**OTHERS**															
30	0.74	0.921	**0.000**	0.877	**0.002**		POS	221.1859	C_10_H_24_N_2_O_3_	221.1860	0.10	104.1071 (100); 60.0808 (1); 58.0657 (1)	Choline derivative	1	
31	15.98	0.765	**0.016**	0.654	0.056		NEG	447.2228	C_21_H_36_O_10_	447.2236	0.80	315.1812 (20); 191.0564 (10); 161.0456 (70); 149.0456 (5); 143.0347 (10); 131.0349 (5); 113.0244 (70); 101.0244 (100)	Geraniol pentoside hexoside (unknow isomer)	1,6	[45]
**UNIDENTIFIED COMPOUNDS**															
32	0.87	0.788	**0.012**	0.683	0.042		NEG	545.1620	ND	-	-	201.0709 (20); 196.0614 (30); 142.0509 (100); 100.0404 (40)	Unknown		
33	1.56	0.877	**0.002**	0.636	0.066		POS	317.1092	C_11_H_16_N_4_O_7_	317.1103	1.10	155.0564 (100)	Unknown		
34	2.16	0.902	**0.001**	0.747	**0.021**		POS	374,1446	C_16_H_23_NO_9_	374.1457	1.10	212.0918 (90); 195.0652 (90); 153.0547 (20); 152.0707 (100); 85.0284 (40); 69.0335 (10)	Unknown		
35	6.46	0.792	**0.011**	0.738	**0.023**		POS	242.0803 (483.1538)	ND	-	-	363.1116 (30); 339.1116 (30); 321.1010 (100); 303.0902 (40)	Unknown (+ hexoside)		
36	6.46	0.846	**0.004**	0.787	**0.012**		POS	490.2646	C_23_H_39_NO_10_	490.2647	0.10	462.0538 (20); 320.0827 (90); 311.0769 (100); 265.1437 (40); 247.1310 (20)	Unknown		
37	6.46	0.854	**0.003**	0.779	**0.013**		POS	942.3170	ND	-	-	499.1261 (10); 378.0767 (80); 320.0706 (100)	Unknown		
38	6.46	0.863	**0.003**	0.772	**0.015**		POS	927.3515	ND	-	-	483.1540 (100); 363.1117 (40)			
39	7.86	0.741	**0.022**	0.708	0.033		NEG	161.0819	C_7_H_14_O_4_	161.0819	0.00	117.0557 (50); 99.0451 (50); 71.0502 (20)	Unknown		
40	11.82	0.748	**0.021**	0.683	0.043		NEG	561.2550	C_26_H_42_O_13_	561.2553	0.30	519.2444 (80); 387.2013 (100); 207.1386 (40); 191.0561 (40); 161.0454 (40); 113.0244 (30); 101.0244 (50); 99.0087 (70); 89.0244 (50); 71.0138 (40)	Unknown		
41	12.38	0.824	**0.006**	0.777	**0.014**		NEG	529.2652	C_26_H_42_O_11_	529.2654	0.20	-	Unknown		
42	14.21	0.888	**0.001**	0.820	**0.007**		NEG	224.0120	C_10_H_8_ClNO_3_	224.0120	0.00	180.0222 (70)	Unknown		

RT: retention time; RI: reliability of the attempted identification (1: main MS/MS fragment ions, 2: UV, 3: NMR, 4: identified in faba beans; 5: identified in other pulses; 6: identified in other plants); ND: not determined.

### 3.3. Non-Volatile Compounds Potentially Responsible for Faba Bean Bitterness

The difference in the bitter intensities has been partly explained by the presence of alkaloids, including vicine and convicine, in faba bean fractions [23]. However, the aim of this research was to gain a better understanding of the non-volatile compounds responsible for bitterness. Thus, linear and logarithmic Pearson correlations (α = 2.5%) were used to determine the relationships between the non-volatile compound areas, in both negative and positive modes, and the bitter intensity of the nine fractions. Indeed, linear regression should be used when the compound concentration is above the threshold, whereas non-linear models are more suitable when its concentration is below the threshold [10,49].

A total of 6 alkaloids, 4 phenolic compounds, 14 amino acids, 3 organic acids, 2 terpenoids, 2 other compounds, and 11 unidentified compounds positively correlated with bitterness were tentatively identified (Table 1). Then, PCA was performed to explore differences in the non-volatile content of the fractions related to bitterness (Figure 2). In the negative mode (Figure 2A), component 1 (47.44%) was more related to the cultivars, whereas component 2 (29.22%) separated the samples among the fractions. Most of the compounds were positively correlated with bitterness according to the linear model alone (6/14) or to both models (8/14). Concerning the positive mode (Figure 2B), component 1 (51.27%) was related to the type of fractions, whereas the cultivars were much more discriminated for component 2 (29.30%). Only one compound (**25**) was positively correlated with bitterness according to the logarithmic model, whereas the majority was highlighted by linear (12/31) or both models (18/31). Unlike the PCA from the negative mode, there were two groups of positively correlated compounds with bitterness, each of which appeared to be dependent on the P1 (bottom right) or P2 fractions (top right). N-acetyl-L-tyrosine hexoside (**10** and **14**) and L-DOPA hexoside derivative (**19**) were more characteristic of P2, whereas P1 was more related to the presence of vicine (**1**) and alkaloid derivatives (**2**, **3**, **4**, **5**, and **6**), L-arginine (**7**) and a derivative (**17**), L-tryptophan (**15**), and L-DOPA (**13**) and its derivatives (**11**, **12**, and **15**).

Among the alkaloids, vicine (1) is known to activate TAS2R16 and should be partly responsible for faba bean bitterness [23]. This suggested that vicine derivatives (**2**, **3**, **4**, and **6**) also activated this receptor, notably if their β-glucopyranoside moiety was free to bind to the active site of TAS2R16 [50]. Concerning amino acids, L-phenylalanine (**16**) has been shown to activate TAS2R1 and 8, whereas L-tryptophan (**18**) only activates TAS2R4 and 39 receptors [51,52]. These results were consistent with the literature, which confirms that this multidisciplinary approach was relevant for identifying potential bitter compounds. In addition, the compound (**15**) was a derivative of L-tryptophan and could therefore activate bitter taste receptors. L-tyrosine, also a hydrophobic amino acid, exhibits a bitter taste [53], which suggested that N-acetyl-tyrosine hexoside (**10** and **14**) was characterised by a bitter taste. L-arginine (**7**) did not activate TAS2Rs, but this amino acid enhanced the bitter intensity of 6-n-propylthiouracil (PROP) and caffeine [54]. This suggested that the presence of free L-arginine in faba bean fractions could increase the bitter perception of the bitter compounds. There was no information on the bitterness of p-hydroxybenzoic hexoside (**21**), protocatechuic acid hexose (**22**), kaempferol derivative (**24**), and kynurenic acid derivative (**26**), but the bitter characteristics of the molecules from which they were derived have been determined. p-Hydroxybenzoic acid and protocatechuic acid were perceived as slightly strong bitter and moderately bitter, respectively, at a concentration of 2 g/L in water [55]. TAS2R14 and 39 receptors have been shown to be sensitive to kaempferol at a concentration in the µM range [56]. The bitter detection threshold of kynurenic acid in distilled water was 78.1 ppm [57]. In addition, it would be interesting to identify the compounds that were negatively correlated with bitterness that would potentially mask bitter perception and behave as potential TAS2R blockers.

These results were consistent with our previous research focused on the role of saponins (DDMP soyasaponin and soyasaponin βb) and alkaloids (vicine and convicine) in faba bean bitterness. Indeed, alkaloids should be partly responsible for bitterness, whereas the soyasaponin concentration was too low to activate TAS2R receptors [23]. In this current study, soyasaponins were detected but were not positively correlated with bitterness. In addition, vicine was also correlated with the bitter taste, which suggested anew the role of this molecule in faba bean off-flavours. Unlike peas, saponins did not contribute to the bitterness of faba beans [10,12].

Most of the tentatively identified compounds were derived from plant defence metabolism. Alkaloids limit the mollusc repellency of lupin plants [58]. It has been shown that water stress promotes the generation of alkaloids in faba beans [59]. Moreover, vicine exhibits fungicidal and insecticidal properties [60]. Amino acids serve mainly as intermediates in the generation of metabolites, but sometimes they have direct defensive functions against herbivores. For example, L-DOPA, a derivative of L-tyrosine, is a precursor or mimic of neurotransmitters that have been associated with insecticidal properties [61]. There was no information on the defence properties of N-acetyl-L-tyrosine hexoside (**10** and **14**), but N-acetyl-L-tyrosine was produced in common beans during fungal pathogen attacks [32]. Phenolic compounds are known to protect plant tissues against UV irradiation and attacks from herbivores, fungi, and viruses [62]. Concerning organic acids, kynurenic acid, which comes from tryptophan metabolism, is involved in plant defence mechanisms [38,40]. In pine, the catabolism of abscisic acid was activated during infection by *Fusarium circinate*, leading to the production of dihydrophaseic acid [63]. There were two hypotheses to explain the bitterness of a compound. The first hypothesis supposed that bitter molecules in plants would repel aggressors (insects, herbivores) to allow their development for survival. The second hypothesis suggests that bitter compounds are toxic to animals and prevent them from ingesting harmful doses [64]. In this article, the first hypothesis could explain the bitter taste of these compounds, which ensures the plant’s healthy development and survival. However, vicine and convicine are dangerous for people suffering from favism [65], and the second hypothesis thus could be plausible for specific compounds to prevent them from being ingested. It should be noted that these molecules, which were potentially responsible for off-flavours, also ensured good agricultural yields and highlighted that cultivars must be carefully selected. Thus, it would be interesting to also identify compounds that were not positively correlated with bitterness and verify if they are also involved in plant defence mechanisms to better select cultivars.

Finally, combining sensory and untargeted metabolomic approaches allowed us to target potential bitter compounds among a high number of metabolites. However, it was possible that some of these compounds were specific to the P1 and P2 fractions without activating the bitter taste receptors. For most compounds, only the chemical class or main moiety from which they were derived was tentatively used to characterise them. It would be interesting to confirm the identification of these compounds by standards and then determine their bitter characteristics by sensory and/or cellular approaches [9]. However, this approach was probably not complete for identifying all the bitter compounds. Indeed, some metabolites could be present in all the samples but insufficient concentrations to be detected. If several molecules were insufficient to activate the same bitter taste receptor alone, it was possible that all the molecules together, in the same sample, activated a TAS2R at the origin of perceived bitterness.

## 4. Conclusions

In this study, a combination of sensory and untargeted metabolomic analyses was used to tentatively identify the potential non-volatile compounds responsible for bitterness in air-classified faba bean fractions. Bitterness was more related to high-alkaloid cultivars and P fractions. A total of 42 compounds were positively correlated with bitterness, including 6 alkaloids, 4 phenolic compounds, 14 amino acids, 3 organic acids, 2 terpenoids, and 2 other compounds. However, the tentative identification of 11 compounds remains to be determined. To our knowledge, this work is the first one illustrating the variety of compounds from faba beans that are strong markers of bitterness. It should be interesting to apply our experimental design and this untargeted metabolomic approach to other faba bean cultivars. These results showed that bitter compounds in faba beans were different from those in peas, whose bitterness was more related to flavonoids and saponins. Then, the majority of these compounds were defence metabolites that were generated in the plants or seeds when they were exposed to abiotic or biotic stresses. This suggested that faba beans produced bitter compounds to prevent them from being ingested by pests to ensure their survival. One strategy for reducing the bitterness of faba beans is to select cultivars with low levels of these compounds while ensuring the presence of other defence metabolites to guarantee sensory, agronomic, and economic benefits. It should also be interesting to confirm the identification of the highlighted compounds to verify their ability to activate TAS2Rs by in vitro cellular-based assays or to be sensory detected by panellists. Finally, this approach allowed us to select potential compounds responsible for faba bean bitterness, but it did not consider the interactions between the compounds in the fractions, which could modulate their bitter intensity. However, this approach was relevant for reducing the number of studied compounds and can be applied to other food matrices or to analyse negative perceptions related to volatile compounds in faba beans such as green, rancid, and metallic notes, for example.

## Data Availability

The data are not publicly available due to confidential reasons.

## Supplementary Materials

The following supporting information can be downloaded at: https://www.mdpi.com/article/10.3390/metabo13080964/s1. Supplementary Materials about the workflow.
