# Supplementary material for: Faba Bean (Vicia faba L. minor) Bitterness: An Untargeted Metabolomic Approach to Highlight the Impact of the Non-Volatile Fraction
Source: Metabolites. 2023 Aug 21;13(8):964. doi: 10.3390/metabo13080964 (PMC10456379; doi:10.3390/metabo13080964)
Supplement: Supplementary file 1 [file metabolites-13-00964-s001.zip › metabolites-2565851-supplementary.pdf]

Faba Bean (*Vicia faba* L. *minor*) Bitterness: An Untargeted Metabolomic Approach to Highlight the Impact of the Non-Volatile Fraction

Adeline Karolkowski [1,2], Emmanuelle Meudec [3,4], Antoine Bruguière [1], Anne-Claire Mitaine-Offer [1], Emilie Bouzidi [5], Loïc Levavasseur [2], Nicolas Sommerer [3,4], Loïc Briand [1,*] and Christian Salles [1,*]

Supplementary Materials about the workflow.

Real samples, QC and blank data were processed by Compound Discoverer™ software (v. 3.2.2.421, Thermo Scientific, USA) with a workflow constituted by several steps: spectrum processing nodes which extracted the mass spectral data from the input set file and align retention of the compounds in the sequence; compound detection nodes which detected compounds by extraction of masses, managed missing values, grouped adduct and isotopic peaks; peak area refinement nodes which applied QC correction and removed background compounds found in the blank samples.

All parameters are the same in positive and negative mode, except for "Processing node 9: Detect Compounds" and "Processing node 31: Group Compounds"

Processing node 33: Select Spectra

----

1. Spectrum Properties Filter:
- Lower RT Limit: 0.3
- Upper RT Limit: 17
- First Scan: 0
- Last Scan: 0
- Ignore Specified Scans: (not specified)
- Lowest Charge State: 0
- Highest Charge State: 3
- Min. Precursor Mass: 130 Da
- Max. Precursor Mass: 1800 Da
- Total Intensity Threshold: 0
- Minimum Peak Count: 1

2. Scan Event Filters:

- Mass Analyzer: (not specified)
- MS Order: Any
- Activation Type: (not specified)
- Min. Collision Energy: 0
- Max. Collision Energy: 1000
- Scan Type: Any
- Polarity Mode: (not specified)

3. Peak Filters:
- S/N Threshold (FT-only): 3

4. Replacements for Unrecognized Properties:
- Unrecognized Charge Replacements: 1
- Unrecognized Mass Analyzer Replacements: ITMS
- Unrecognized MS Order Replacements: MS^2
- Unrecognized Activation Type Replacements: CID
- Unrecognized Polarity Replacements: +
- Unrecognized MS Resolution@200 Replacements: 60000
- Unrecognized MS^n Resolution@200 Replacements: 30000

5. General Settings:
- Precursor Selection: Use $MS^{(n-1)}$ Precursor
- Use Isotope Pattern in Precursor Reevaluation: True
- Provide Profile Spectra: Automatic
- Store Chromatograms: False

Processing node 26: Align Retention Times
----
1. General Settings:
- Alignment Model: Adaptive curve
- Alignment Fallback: None
- Maximum Shift [min]: 1
- Shift Reference File: True
- Mass Tolerance: 2 ppm
- Remove Outlier: True

Processing node 9: Detect Compounds
----
1. General Settings <u>(negative mode)</u>:

- Mass Tolerance [ppm]: 1 ppm
- Intensity Tolerance [%]: 50
- S/N Threshold: 2
- Min. Peak Intensity: 5000000
- Ions:
    $[2M+FA-H]^{-1}$
    $[2M-H]^{-1}$
    [M+Cl]-1
    $[M+FA-H]^{-1}$
    $[M-2H]^{-2}$
    $[M-H]^{-1}$
- Base Ions: $[M-H]^{-1}$
- Min. Element Counts: C H O
- Max. Element Counts: C_{190} H_{190} N_8 O_{120} S_2

1. General Settings <u>(positive mode)</u>:
- Mass Tolerance [ppm]: 1 ppm
- Intensity Tolerance [%]: 50
- S/N Threshold: 2
- Min. Peak Intensity: 15000000
- Ions:
    $[2M+H]^{+1}$
    $[M+2H]^{+2}$
    $[M+3H]^{+3}$
    $[M+H]^{+1}$
    $[M+K]^{+1}$
    $[M+Na]^{+1}$
    $[M+NH4]^{+1}$
- Base Ions: $[M+H]^{+1}$
- Min. Element Counts: C H O
- Max. Element Counts: C_{190} H_{190} N_8 O_{120} S_2

2. Peak Detection:
- Filter Peaks: True
- Max. Peak Width [min]: 0.6
- Remove Singlets: True
- Min. # Scans per Peak: 5
- Min. # Isotopes: 2

3. Isotope Grouping:

- Min. Spectral Distance Score: 0
- Remove Potentially False Positive Isotopes: True

Processing node 31: Group Compounds
----
1. Compound Consolidation:
- Mass Tolerance: 1 ppm
- RT Tolerance [min]: 0.6

2. Fragment Data Selection (negative mode):
- Preferred Ions: $[M-H]^{-1}$

2. Fragment Data Selection (positive mode):
- Preferred Ions: $[M+H]^{+1}$

Processing node 66: Fill Gaps
----
1. General Settings:
- Mass Tolerance: 2 ppm
- S/N Threshold: 2
- Use Real Peak Detection: True

Processing node 67: Apply Missing Value Imputation
----
1. General Settings:
- Imputation Method: Median + Small Value with Variability
- Fill Blanks with min value: False

2. Random Forest Settings:
- Number of trees: 100
- Max Number of Iterations: 10

Processing node 68: Apply QC Correction
----
1. General Settings:
- Regression Model: Linear

- Min. QC Coverage [%]: 40
- Max. QC Area RSD [%]: 30
- Max. Corrected QC Area RSD [%]: 25
- Max. # Files Between QC Files: 15

Processing node 71: Normalize Areas

----

1. General Settings:

- Normalization Type: Constant Median
- Exclude Blanks: True

Processing node 69: Mark Background Compounds

----

1. General Settings:

- Max. Sample/Blank: 5
- Max. Blank/Sample: 0
- Hide Background: True
